# Acquired resistance to osimertinib in patients with non-small-cell lung cancer: mechanisms and clinical outcomes

**DOI:** 10.1007/s00432-020-03239-1

**Published:** 2020-05-08

**Authors:** Yuxin Mu, Xuezhi Hao, Puyuan Xing, Xingsheng Hu, Yan Wang, Teng Li, Jinyao Zhang, Ziyi Xu, Junling Li

**Affiliations:** Department of Medical Oncology, National Cancer Center/National Clinical Research Center for Cancer/Cancer Hospital, Chinese Academy of Medical Sciences and Peking Union Medical College, Number 17 Panjiayuan Nan Li, Chao Yang District, Beijing, 100021 China

**Keywords:** Non-small-cell lung cancer, Osimertinib, T790M, Resistance mechanism

## Abstract

**Purpose:**

Osimertinib, a third-generation epidermal growth factor receptor tyrosine-kinase inhibitor (EGFR-TKI), has demonstrated substantial clinical benefit in patients with non-small-cell lung cancer (NSCLC) who were resistant to early-generation EGFR-TKIs and had acquired a T790M mutation. The aim of our study was to identify the mechanisms underlying resistance to osimertinib and to correlate them with clinical outcomes.

**Methods:**

We retrospectively analyzed patients with advanced NSCLC who received osimertinib for T790M-mutated acquired resistance to prior EGFR-TKIs between March 1, 2017 and December 31, 2018. Patients with paired molecular data of pre-osimertinib and after resistance development, which were not confirmed with small-cell lung cancer (SCLC) transformation, were included in the molecular analysis set.

**Results:**

Of 49 patients evaluated in the molecular analysis set, 24 patients maintained T790M mutation, while 25 patients exhibited T790M-loss. Molecular modifications were identified in 27 of 49 patients including EGFR acquired mutations (C797S, C796S, G796S, V802I, V834L, E758D and G724S), non-EGFR-dependent mutations (PIK3CA, ALK, BRAF, KRAS and TP53), EGFR amplification and MET amplification. At data cutoff, median progression-free survival (PFS) was 9.3 months in the T790M-retain group compared with 7.8 months in T790M-loss patients (*P* = 0.053). Median PFS was significantly longer in patients with EGFR-dependent resistance mechanism (13.5 months) than in those with alternative pathway activation (8.2 months; *P* = 0.012).

**Conclusions:**

The study revealed heterogeneous mechanisms of resistance to osimertinib in advanced NSCLC patients and their association with clinical outcomes. Patients who maintained T790M mutation or with EGFR-dependent resistance mechanism had longer clinical outcome benefits.

## Introduction

Lung cancer is the leading cause of cancer-related deaths worldwide (Siegel et al. 2015). The discovery of small-molecule anti-cancer drugs, which target specific oncogenic driver mutations, dramatically changed the clinical therapeutic modality for patients with non-small-cell lung cancers (NSCLC). The most common oncogenic driver mutation in lung cancer is epidermal growth factor receptor (EGFR) mutation, which characterizes approximately 40–55% of all NSCLC in Asian patients (Shi et al. 2014). Numerous randomized trials have demonstrated the superiority of small-molecule EGFR tyrosine-kinase inhibitors (TKIs) over cytotoxic chemotherapy for the treatment of EGFR-mutant NSCLC patients and, thus, have been established as standard first-line therapy (Mok et al. 2009; Rosell et al. 2012; Sequist et al. 2013). Most patients who were treated with first- or second-generation EGFR-TKIs developed acquired resistance after about 8–14 months, and approximately 50–60% of patients had a T790M acquired resistance mutation (Yu et al. 2013; Lee et al. 2019). Osimertinib is a potent, irreversible, third-generation EGFR-TKI, which inhibits both EGFR-activating and T790M resistance mutations (Cross et al. 2014). The AURA3 phase III trial showed that osimertinib had a superior performance to standard platinum-based chemotherapy in terms of the objective response rate (ORR), progression-free survival (PFS) and tolerability in patients with T790M-mediated acquired resistance (Mok et al. 2017). Osimertinib has been approved for the treatment of NSCLC patients carrying a T790M resistance mutation after disease progression of prior EGFR-TKI therapy. Various resistance mechanisms of osimertinib have been reported including EGFR modifications, alternative pathway activation or histological transformation, but these are not yet fully understood (Oxnard et al. 2018). The prognostic significance of different resistance mechanisms has not been well documented in the literature. The aim of our study was to elucidate the mechanisms of acquired resistance to osimertinib and to explore the association of clinical outcomes with various genetic modifications.

## Materials and methods

### Study population

**Fig. 1:**
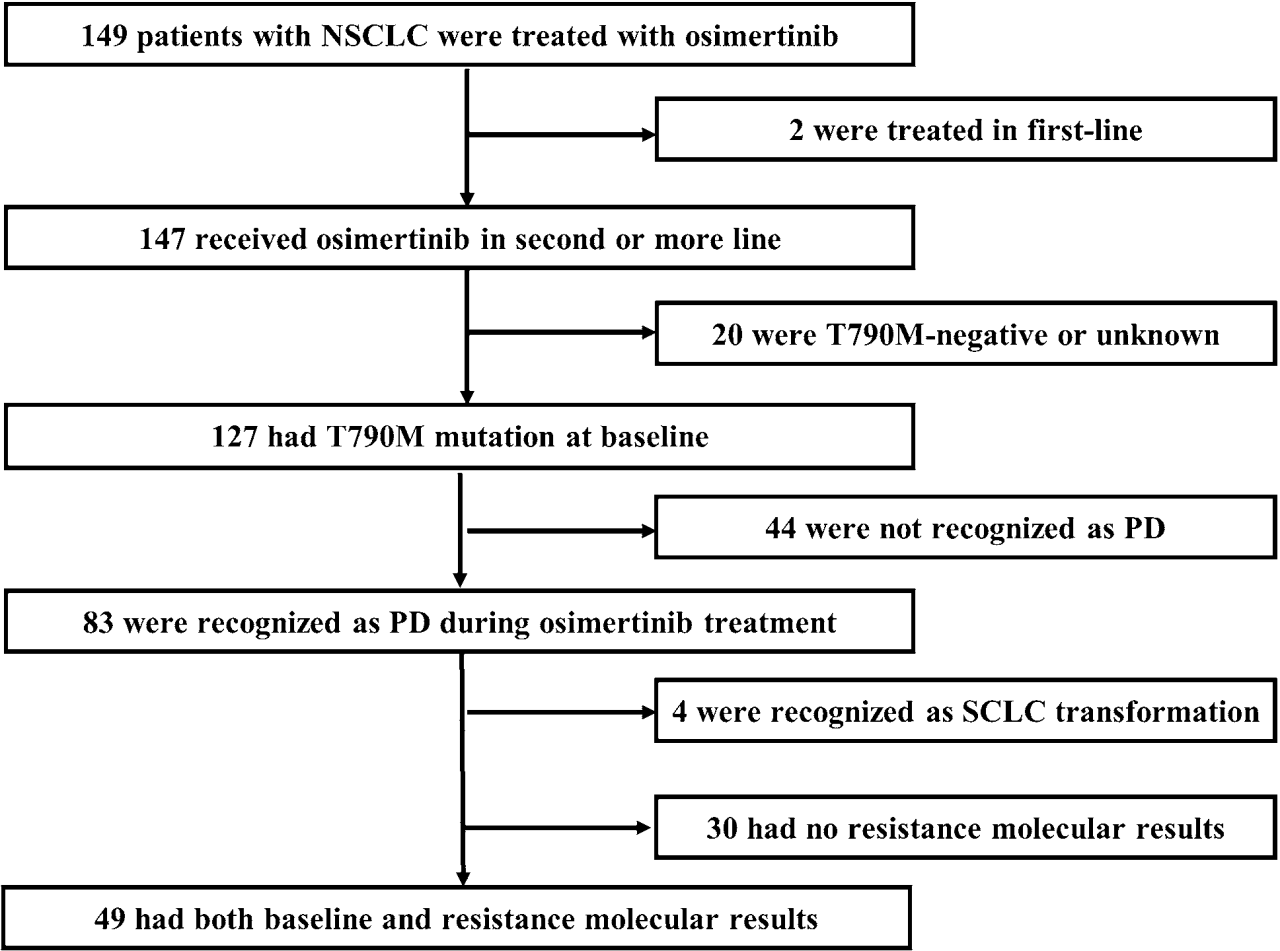
Flowchart of patient selection in the molecular analysis set. *NSCLC* non-small-cell lung cancer, *PD* progressive disease, *SCLC* small-cell lung cancer

Between March 1, 2017 and December 31, 2018, patients who received osimertinib were retrospectively identified in our cancer center. Inclusion criteria included histologically or cytologically confirmed NSCLC, advanced stage (including stages IIIB and IV), acquired resistance to prior EGFR-TKI therapy and harboring T790M resistance mutation in either tumor or plasma samples before receiving single-agent osimertinib therapy. Patients who received osimertinib for less than 3 weeks for any reason were excluded from the study. Patients with paired molecular information before osimertinib initiation and after progression, and who were not confirmed with small-cell lung cancer (SCLC) transformation were included in the molecular analysis set. A flowchart outlining the selection of 49 patients in the molecular analysis set is presented in Fig. 1. We retrospectively collected demographic and clinical characteristics of patients including sex, age, smoking history, histology, Eastern Cooperative Oncology Group performance status (ECOG PS), treatment history, metastases sites prior to osimertinib, osimertinib treatment procedure and molecular information. The data cutoff for analysis was May 14, 2019.

### Assessments

The primary aim of the study was to characterize the mechanisms of resistance to osimertinib in patients previously treated for NSCLC. Plasma and tumor samples of pre-osimertinib and after radiological identified progression were mostly tested using next-generation sequencing (NGS) panels (168-gene panel), according to their medical records, and paired molecular results were analyzed. The clinical aim was to explore the association of clinical outcomes with various genetic modifications. Clinical response was investigated according to Response Evaluation Criteria in Solid Tumors (RECIST) version 1.1. Disease control rate (DCR) was defined as the percentage of patients who had complete response (CR), partial response (PR) or stable disease (SD). PFS was defined as the duration from the start of osimertinib therapy until progressive disease (PD) or death from any cause. The treatment duration (TD) was measured from the start of osimertinib until the last dose for any reason.

### Statistical analysis

Statistical analyses were carried out using SPSS 23.0 statistical software (SPSS, Inc., Chicago, IL, US). Survival analyses were performed using the Kaplan–Meier method and compared using a log-rank test between groups. A chi-squared test and Fisher’s exact test were used for comparing DCR. All *P* values were two sided and a *P* value < 0.05 was considered to be statistically significant.

## Results

### Patients and characteristics

We collected baseline molecular data from 53 patients treated with osimertinib who had acquired a T790M resistance mutation to first- or second-generation EGFR-TKIs. All patients were adenocarcinomas at baseline and received osimertinib 80 mg once daily. After resistance to osimertinib became apparent, 21 patients underwent re-biopsies, in which 4 patients were confirmed with a SCLC transformation. Forty-nine patients who were not confirmed with a histology transformation, and with paired molecular data of pre-osimertinib and after osimertinib resistance, were included in the molecular analysis set. Patient demographics and baseline characteristics of the molecular analysis set are presented in Table 1. Twenty-five patients received osimertinib as second line, the other 24 patients were third or later line. Median age was 59 years, 61.2% of patients were women, 77.6% were non-smokers and 95.9% had an ECOG PS of 0 or 1. EGFR-T790M/19del-positive and T790M/L858R-positive were detected in 30 (61.2%) and 17 (34.7%) patients at baseline, respectively. Prior to osimertinib, EGFR-TKI treatment history included gefitinib (25/49, 51.0%), erlotinib (10/49, 20.4%), icotinib (18/49, 36.7%), afatinib (1/49, 2.0%) and avitinib (3/49, 6.1%). Lung (38/49, 77.6%), bone (28/49, 57.1%), pleural (19/49, 38.8%) and central nervous system (CNS) (18/49, 36.7%) metastases were common metastatic sites prior to osimertinib. All patients in molecular analysis set underwent genetic testing at least three times during course of disease: before first EGFR-TKI treatment, pre-osimertinib and after osimertinib resistance. Samples for genetic testing before first EGFR-TKI treatment were mostly tumor tissues (43/49, 87.8%); while, samples used for pre-osimertinib and after osimertinib resistance testing were both mostly plasma (34/49, 69.4%).

**Table 1:** Baseline patient demographic and clinical characteristics of the molecular analysis set

Characteristics	Patients (*n* = 49)	
	No	%
Age (years)		
Median	59	
Range	42–84	
Sex		
Male	19	38.8
Female	30	61.2
ECOG PS		
0	27	55.1
1	20	40.8
2	2	4.1
Smoking status		
Non-smoker	38	77.6
Former/current smoker	11	22.4
Histology		
Adenocarcinoma	48	98.0
Others	1	2.0
Genotypes		
T790M-positive		
Exon 19del-positive	30	61.2
L858R-positive	17	34.7
Exon 19del/ L858R-negative	2	4.1
Osimertinib treatment line		
2nd	25	51.0
≥ 3rd	24	49.0
Treatment history		
Gefitinib	25	51.0
Erlotinib	10	20.4
Icotinib	18	36.7
Afatinib	1	2.0
Avitinib	3	6.1
Chemotherapy	25	51.0
Metastases		
Lung	38	77.6
Bone	28	57.1
Pleural	19	38.8
CNS	18	36.7
Liver	6	12.2
Adrenal gland	6	12.2

*CNS* central nervous system, *ECOG PS* Eastern Cooperative Oncology Group performance status

### Clinical outcomes

Of 49 patients in the molecular analysis set, baseline pre-osimertinib molecular analysis showed that 30 patients had T790M + /19del + , 17 had T790M + /L858R + and 2 had T790M + /19del-/L858R-. The median PFS for osimertinib was 9.0 months (95% CI 8.2, 9.8) and varied across genotypes. In patients with T790M + /19del + , the median PFS was 9.3 months (95% CI 1.5, 17.1), which was significantly longer than patients with T790M + /L858R + (median: 8.5 months [95% CI 6.3, 10.7]) (*P* = 0.005). A total of 45 patients were available for evaluation of the disease response in these two genotypes. The DCR was 100% (29/29) in patients with T790M + /19del + and 93.8% (15/16) in patients with T790M + /L858R + (*P* = 0.356).

**Fig. 2:**
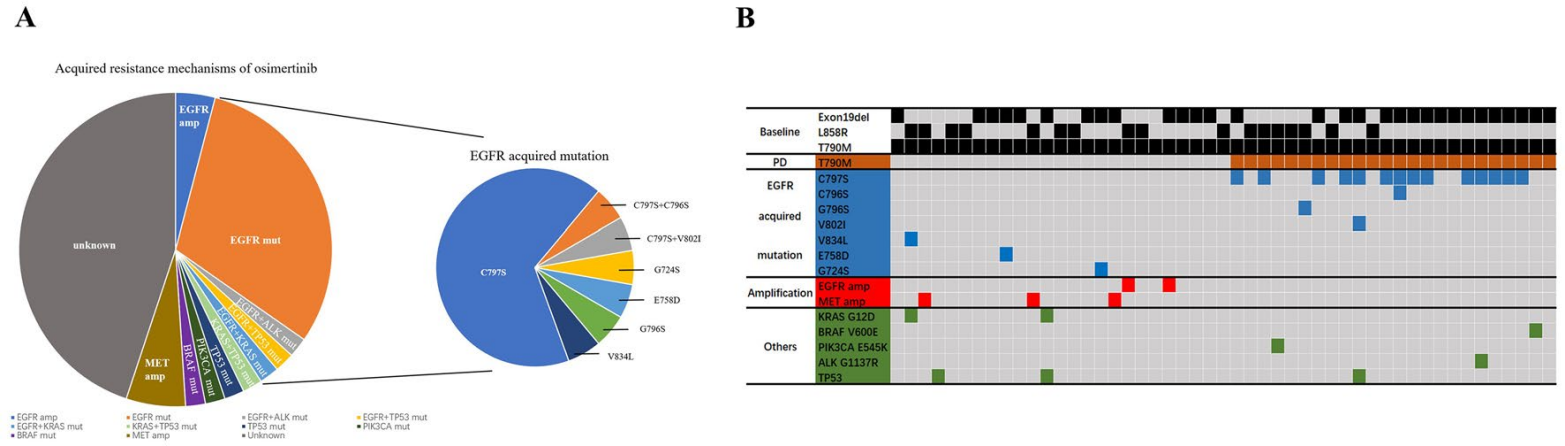
Molecular modification after acquired resistance to osimertinib

In patients who developed resistance to osimertinib, possible genomic resistance mutations were identified in 27 of 49 patients in the molecular analysis set. Fourteen patients (14/49, 28.6%) acquired secondary EGFR C797S mutation, all in *cis* with the initial EGFR exon19del/L858R mutation and the T790M mutation. Other EGFR-dependent molecular modifications included C796S mutation (1/49, 2.0%), G796S mutation (1/49, 2.0%), V802I mutation (1/49, 2.0%), V834L mutation (1/49, 2.0%), E758D mutation (1/49, 2.0%), G724S mutation (1/49, 2.0%) and EGFR amplification (2/49, 4.1%). Non-EGFR modifications were identified in 10 patients (10/49, 20.4%) including activating mutations of PIK3CA (1/49, 2.0%), ALK (1/49, 2.0%), BRAF (1/49, 2.0%), KRAS (2/49, 4.1%), TP53 (3/49, 6.1%) and MET amplification (3/49, 6.1%). Nineteen patients had no new findings in osimertinib resistance testing except for pre-existing EGFR mutations pre-osimertinib, among whom 12 patients showed T790M-loss. Negative NGS results of plasma ctDNA samples were found in the remaining three patients. A summary of resistance mechanisms to osimertinib are shown in Fig. 2. Patients with an EGFR-dependent resistance mechanism more likely occurred in the base of exon 19del (53.3% in exon19del vs 17.6% in L858R, *P* = 0.017); while, alternative pathway activation showed a trend to present in the base of the L858R mutation (10.0% in exon19del vs 23.5% in L858R, *P* = 0.409).

**Fig. 3:**
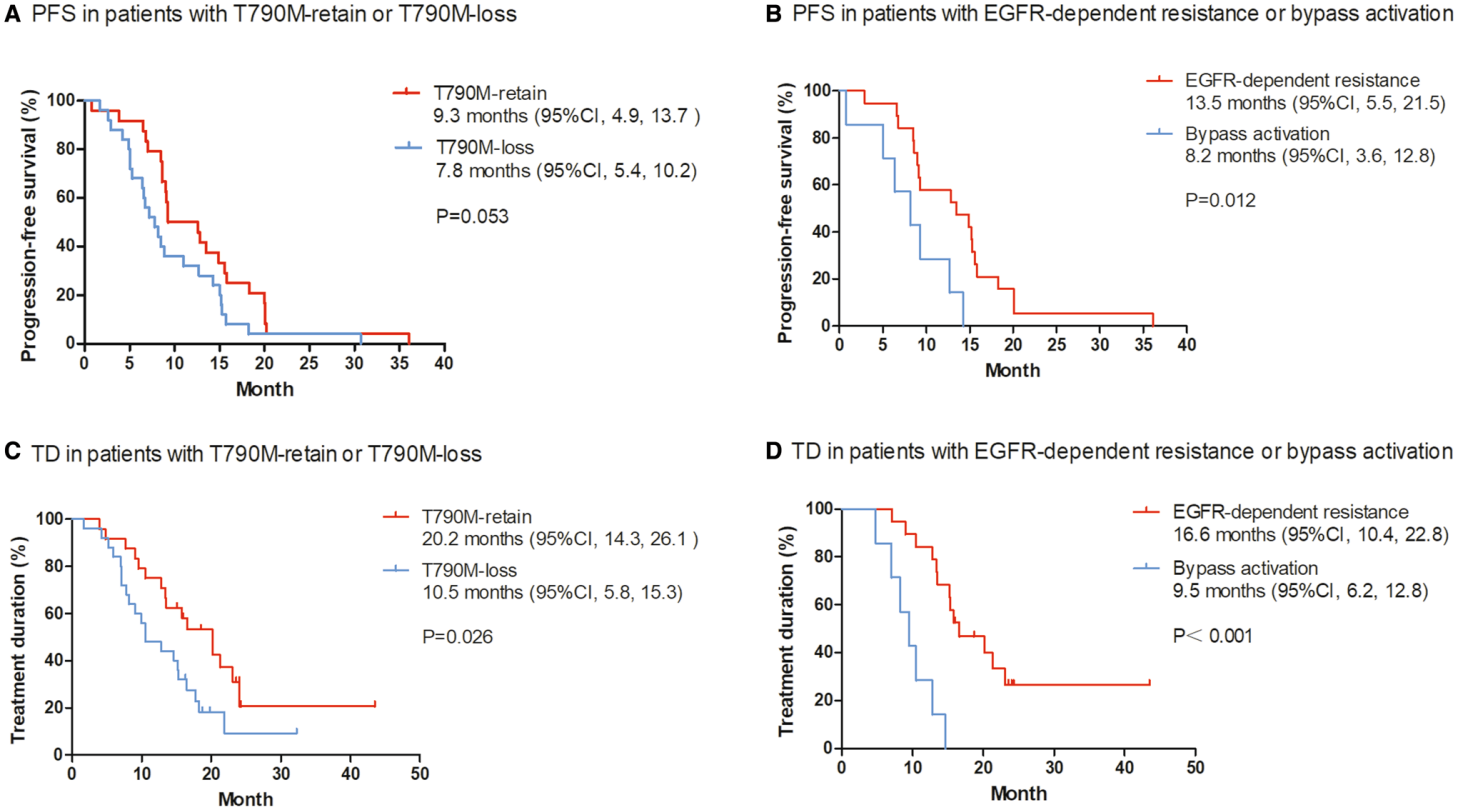
PFS in patients with T790M-retain or T790M-loss after progression of osimertinib therapy (**a**), in patients with EGFR-dependent resistance mechanism or bypass activation after progression (**b**). TD in patients with T790M-retain or T790M-loss after progression of osimertinib (**c**), in patients with an EGFR-dependent resistance mechanism or bypass activation after progression (**d**). *PFS* progression-free survival, *TD* treatment duration, *CI* confidence interval. Tick marks indicate censored observations

**Fig. 4:**
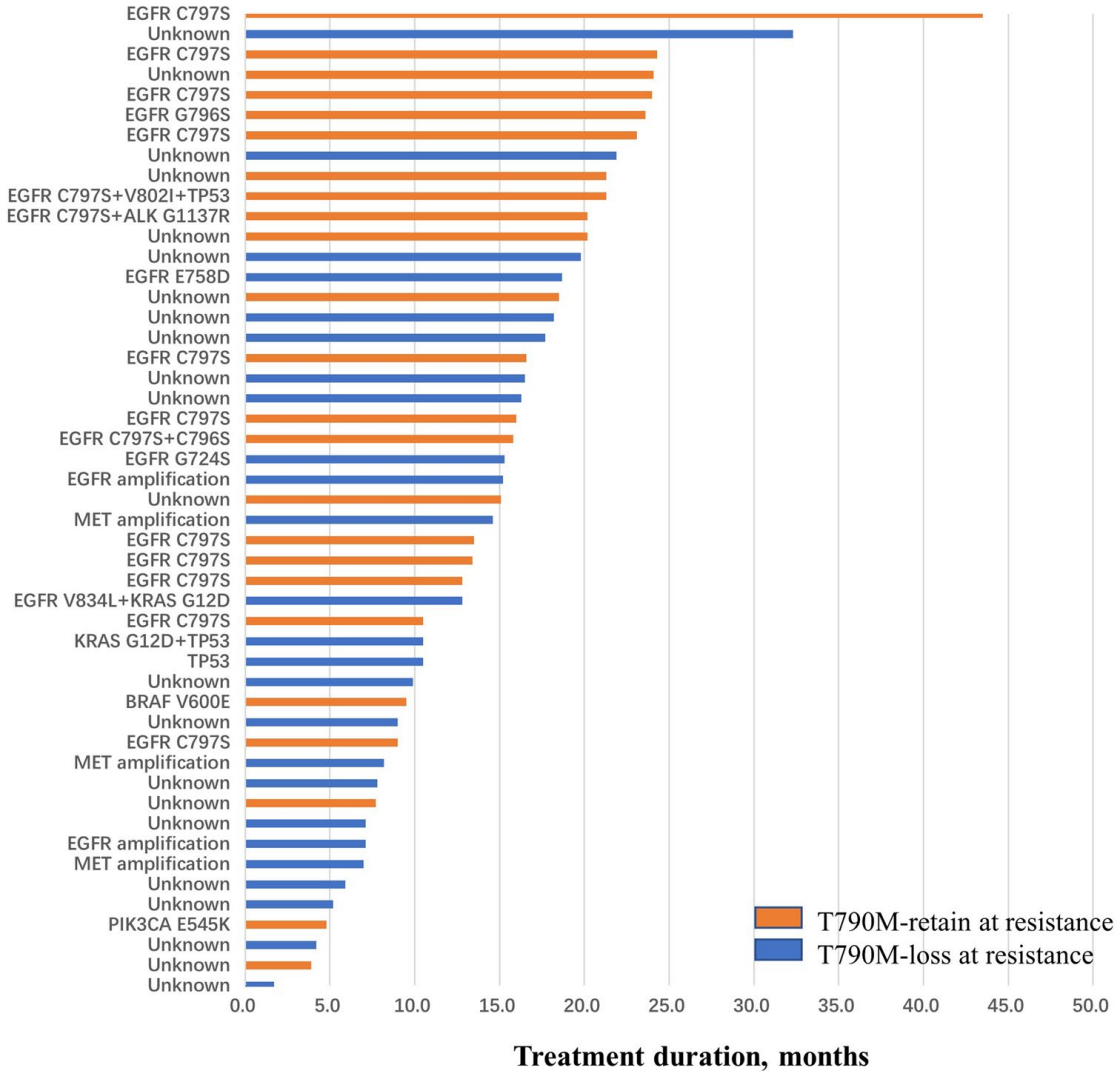
Treatment duration in 49 patients in the molecular analysis set

T790M-loss was the most frequent phenomenon in the molecular analysis set, observed in 25 (51.0%) patients and T790M-retain was found in 24 patients (49.0%). We explored the prognosis difference between patients with T790M-retain and T790M-loss after progression with osimertinib therapy. Patients with T790M-retain showed a trend of longer PFS, but this was not statistically significant compared to patients with T790M-loss (median PFS, 9.3 months [95% CI 4.9, 13.7] vs 7.8 months [95% CI 5.4, 10.2], *P* = 0.053). After disease progression, 29 (59.2%) of 49 patients continued osimertinib with additional clinical benefit for a median period of 4.2 months (range 1.0–16.3), including 16 patients in the T790M-retain group and 13 patients in the T790M-loss group. The TD of osimertinib was 20.2 months (95% CI 14.3, 26.1) for the T790M-retain group, which was significantly longer than 10.5 months (95% CI 5.8, 15.3) of patients with T790M-loss (*P* = 0.026). We also explored clinical outcome differences between patients who had EGFR-dependent resistance mechanisms and patients with alternative pathway activation. Alternative pathway activation was identified in seven patients including PIK3CA, BRAF and KRAS mutations and MET amplification. Compared with T790M-retain, alternative pathway activation was more likely to found in T790M-loss patients (8.3% vs 20.0%, *P* = 0.448). Patients with alternative pathway activation after osimertinib resistance had shorter PFS and TD than patients with EGFR-dependent resistance mechanisms (median PFS, 8.2 months [95% CI 3.6, 12.8] vs 13.5 months [95% CI 5.5, 21.5], *P* = 0.012; median TD, 9.5 months [95% CI 6.2, 12.8] vs 16.6 months [95% CI 10.4, 22.8], *P* < 0.001) (Figs. 3, 4).

Outside the molecular analysis set, four patients with primary lung adenocarcinoma showed SCLC transformation after osimertinib resistance. Among the SCLC transformation population, three were men and heavy smokers, one was woman and a non-smoker. The original EGFR mutation in one patient was retained except for T790M in the plasma ctDNA sample. The other three patients did not receive gene testing as genotype transformation was detected. These four patients progressed on osimertinib with times that ranged from 1.4 to 5.6 months. The treatment for four SCLC transformation patients was a switch to etoposide/irinotecan plus platinum chemotherapy.

## Discussion

Drug resistance represents a major challenge in targeted cancer therapy. Several studies have reported mechanisms of resistance to third-generation EGFR-TKI osimertinib including C797S mutation, EGFR amplification, bypass activation, phenotypic transformation and so on (Ortiz-Cuaran et al. 2016; Oxnard et al. 2018). Our data add to these findings, providing additional Chinese data setting, the associations of various molecular modifications, and clinical outcomes for possible resistance mechanisms.

Twenty-seven patients had putative genomic resistance mechanisms identified. We found the EGFR C797S mutation to be the most common resistance mechanism to osimertinib in our study, and all were concurrent in cis with T790M mutation. This mutation was detected in approximately 20–30% of osimertinib acquired resistance cases and was resistant to multiple EGFR-TKIs including gefitinib, erlotinib, afatinib and osimertinib when C797S performed in *cis* with a T790M mutation (Niederst et al. 2015; Thress et al. 2015; Sullivan and Planchard 2016). The combination of afatinib and cetuximab demonstrated promising clinical activity and a manageable safety profile in patients who developed acquired resistance to EGFR-TKIs, regardless of the T790M mutation status (Janjigian et al. 2014), which may conquer such EGFR triple mutations. The research of treatment strategies for other EGFR-dependent resistance mechanisms was lacking and needs further investigation. The combination of chemotherapy, bevacizumab and atezolizumab in the IMpower 150 study was promising for the EGFR mutated, prior EGFR-TKI failed patients (Reck et al. 2019). EGFR-independent resistance mechanisms were mainly bypass activation and mostly were mutually exclusive with the T790M mutation, suggesting the possibility of combination therapy. MET amplification is a common mechanism of resistance with an incidence of 10–30% (Oxnard et al. 2018; Wang et al. 2018). The combination of EGFR-TKI and crizotinib was reported effective against the acquired MET amplification after progression of osimertinib therapy in the clinical setting (Wang et al. 2018). Activation of the RAS-MAPK pathway, such as KRAS mutation and BRAF mutation, was also reported. A combination of MEK or BRAF inhibitors may rescue such mechanism after resistance to osimertinib has developed (Eberlein et al. 2015; Ho et al. 2017). A negative NGS outcome after osimertinib resistance was detected in three patients in our study, which may result from the relatively low tumor burden, with low levels of ctDNA in plasma samples.

T790M-loss and T790M-retain were two basic modes at the time of PD. Loss of T790M was observed in over half of the patients in our study as previously reported (Oxnard et al. 2018), mostly concurrent with the development of bypass activation, and might be associated with earlier resistance to osimertinib. We found that T790M-retain patients had better clinical outcomes to osimertinib including PFS and TD than those with T790M-loss. As patients with T790M-loss are more likely to be associated with the development of bypass activation, the patient cohort with bypass activation in our study had similarly worse outcome than patients with an EGFR-dependent resistance mechanism. The association of bypass activation with the clinical outcome to osimertinib therapy has not previously been reported in detail. Yong He et al. analyzed the correlation of MET amplification and survival outcomes, and found that MET amplification was associated with shorter PFS and overall survival (OS) than in those patients without it after osimertinib progression (Wang et al. 2018). The clinical outcomes of bypass activation need further exploration.

Outside the molecular analysis set, four patients had SCLC transformation after osimertinib resistance. SCLC transformation has been reported as a mechanism of acquired resistance in 3 ~ 14% of patients progressing from first- to third-generation EGFR-TKIs (Sequist et al. 2011; Yu et al. 2013). After the confirmation of SCLC transformation, platinum-etoposide regimen is recommended (Marcoux et al. 2019).

There were several limitations to our study. First, the study was limited to a single center, its retrospective design and small sample size. Second, molecular data of both tissue and plasma samples, whichever were available, were collected for molecular analysis, which may bias the results of the molecular modifications. Future tissue-based analyses are needed to provide a more comprehensive profile of the mechanisms underlying resistance to osimertinib.

In conclusion, molecular re-analysis after osimertinib failure has significant clinical utility for guiding personalized subsequent treatment selection. Further studies on novel or combination therapy are needed to overcome the resistance to osimertinib.

## Data Availability

The data are not publicly available due to privacy and ethical restrictions.
